# Role of MRI in the diagnosis and prognosis of ventricular arrhythmias

**DOI:** 10.1186/1532-429X-13-S1-P250

**Published:** 2011-02-02

**Authors:** Nakul C Sharma, Ian Paterson

**Affiliations:** 1 Univesrity of Alberta-Mazankowski Heart Institute, Edmonotn, AB, Canada

## Background

Many patients with malignant cardiac arrhythmias’ require thorough investigations to determine the etiology. Conventional tests (ECG and echocardiography) often cannot detect a structural basis for the arrhythmia and physicians therefore lack guidance as to the most appropriate treatment.

Previous studies have shown MRI is a more sensitive and specific modality to better elucidate cardiac abnormalities and provide insight to the underlying etiology and better predict clinical outcomes.

## Hypothesis

Compared to conventional testing, Cardiac MRI has incremental benefit in the diagnosis and treatment of patients with ventricular tachycardia and in the assessment/treatment of patients with suspected ARVD.

## Methods

We performed a retrospective review of patients who have undergone CMR as part of their treatment/workup for ventricular arrhythmias and/or ARVD at the University of Alberta Hospital over the last 4 years. CMR results were correlated to patient outcomes: death, cardiac death, hospital admissions, ICD implantation, and electrophysiologic ablation

## Results

A total of 211 patients were enrolled; however complete clinical data on only 142 patients was obtained. The baseline age/sex was 46 years & 59% male. The average BSA was 1.86m^2. Known CAD was documented in 9.8%.

The presenting symptom(s) to medical attention are as follows: Syncope 36.6%, Palpitation 36.6%, Arrest 9.2%, None 17.6%. The arrhythmia’s documented upon patient review were Atrial Tachycardia 2.8%, 10.5%, NSVT 28.8%, VT 31.7%, and VF 5.6%, None 20.6%)

Based on the clinical presentation 55% of patients were admitted to hospital, 37% went on to have an EPS procedure. There were 12 cardiac arrests at presentation that received an ICD. A further 6% who did not arrest also received an ICD

The corresponding Ejection fractions can be seen in Table 1.

**Table 1:** Average Ejection fraction based on Imaging Modality

	Echo	CMR	Atypical Gadolinium uptake
LVEF%	52.82	60.36%	58.2%

RVEF%	Normal	55.2%	53%

In terms of ARVD, a diagnosis was made in 2.1% of the patients via CMR while 8.4% of the patients possessed one minor criterion. No diagnosis of ARVD was made on echocardiography or EKG

## Conclusion

CMR’s increased sensitivity is able to detect many more abnormalities but these rarely translate into changes in clinical practice unless in the context of a diagnosis of ARVD. Although a normal CMR is reassuring and likely precludes a favorable outcome 1/3 of patients still underwent further EPS testing with ½ of those resulting in no further intervention. This details that CMR may be a more sensitive tool to exclude malignant causes of ventricular arrhythmia but this has not changed current practice patterns.

